# Managing Potato Biodiversity to Cope with Frost Risk in the High Andes: A Modeling Perspective

**DOI:** 10.1371/journal.pone.0081510

**Published:** 2014-01-30

**Authors:** Bruno Condori, Robert J. Hijmans, Jean Francois Ledent, Roberto Quiroz

**Affiliations:** 1 Liaison Office in Bolivia, International Potato Center, La Paz, La Paz, Bolivia; 2 Department of Environmental Science and Policy, University of California, Davis, Davis, California, United States of America; 3 Unité d’Ecophysiologie et d’Amélioration Végétale, Université Catholique de Louvain, Louvain-la-Neuve, Brabant, Belgium; 4 Integrated Crops and Systems Research Program, International Potato Center, La Molina, Lima, Peru; Key Laboratory of Horticultural Plant Biology (MOE), China

## Abstract

Austral summer frosts in the Andean highlands are ubiquitous throughout the crop cycle, causing yield losses. In spite of the existing warming trend, climate change models forecast high variability, including freezing temperatures. As the potato center of origin, the region has a rich biodiversity which includes a set of frost resistant genotypes. Four contrasting potato genotypes –representing genetic variability- were considered in the present study: two species of frost resistant native potatoes (the bitter *Solanum juzepczukii*, var. Luki, and the non-bitter *Solanum ajanhuiri*, var. Ajanhuiri) and two commercial frost susceptible genotypes (*Solanum tuberosum* ssp. *tuberosum* var. Alpha and *Solanum tuberosum* ssp. *andigenum* var. Gendarme). The objective of the study was to conduct a comparative growth analysis of four genotypes and modeling their agronomic response under frost events. It included assessing their performance under Andean contrasting agroecological conditions. Independent subsets of data from four field experiments were used to parameterize, calibrate and validate a potato growth model. The validated model was used to ascertain the importance of biodiversity, represented by the four genotypes tested, as constituents of germplasm mixtures in single plots used by local farmers, a coping strategy in the face of climate variability. Also scenarios with a frost routine incorporated in the model were constructed. Luki and Ajanhuiri were the most frost resistant varieties whereas Alpha was the most susceptible. Luki and Ajanhuiri, as monoculture, outperformed the yield obtained with the mixtures under severe frosts. These results highlight the role played by local frost tolerant varieties, and featured the management importance –e.g. clean seed, strategic watering- to attain the yields reported in our experiments. The mixtures of local and introduced potatoes can thus not only provide the products demanded by the markets but also reduce the impact of frosts and thus the vulnerability of the system to abiotic stressors.

## Introduction

The Altiplano is a high tropical plateau located at 3600–4300 m above sea level in the Andes of Bolivia and Peru. Most of the cropland is located below 4000 masl; above that elevation land is mainly covered by natural grasslands and is only used for growing bitter potato landraces, which are adapted to cold conditions. Potato is by far the most important crop in the region, accounting for 44% of the gross value of crop production [1] from a cropping area of about 88,000 ha [2]. Potato production is limited by abiotic and biotic factors; Andean farmers manage these constraints mainly by the use of a high diversity of native species and cultivars that are often grown as mixtures in single plots [3], [4], [5]. As potato originated in the Andes [6], local genetic diversity in cultivated potato is large and includes several species, comprising both bitter -*Solanum juzepckzukii* (triploid), and l non-bitter frost resistant potatoes: Solanum *ajanhuiri* (diploid), but also the non-bitter frost susceptible conventional *Solanum tuberosum* subspecies *tuberosum* (tetraploid), and *Solanum tuberosum* ssp. *andigenum* (tetraploid), which are present in the Altiplano [7], [8].

The principal role played by the diversity of potatoes grown in the Altiplano is related to smallholder’s food security. Potato fresh yields in the area are low. In Peru and the northernmost part of the Bolivian Altiplano, yield average varies from 4 to 5.2 t/ha whilst in the southern Bolivian section [1] the average yield is 3.6 t/ha. The growing season in the Altiplano extends from October to March, when maximum annual temperature coincides with the rainy season. In the agricultural zones of the Altiplano, average maximum temperature is around 18°C whereas minimum temperature is around 4°C during the growing season. Precipitation is around 800 mm/year in the northeast of the Altiplano whereas in the southwestern Altiplano, it is about 200 mm/year, mostly occurring during the same growing season. Production risk for potato is high due to several recurrent factors, particularly drought, hail, and frost. Frost-free period averages 140 days in the northern Altiplano and 110 days in the Southern areas [9]. The high production risks presented by frost and other factors may also lead to reduced investment in agriculture, resulting in low production which in turn affects food availability.

The varieties of the species *Solanum tuberosum* ssp. *andigenum* are the most widely cultivated in the Andes. The *Solanum juzepczukii* stands out for its high frost and drought tolerance and its capacity to grow at 4000 masl and above [10]. However its tubers are bitter due to a high content of glycoalkaloids, requiring processing for direct human consumption [11]. This processing is an old Andean’s strategy for conserving food – chuño: dehydrated potatoes - for several years [12]. It has been estimated that at least 25% of total area under potato in the Altiplano is planted with bitter varieties [13]. This assertion is supported by the estimate that bitter potatoes make up 15% of total potato area in Bolivia [11]. In fact, more bitter potatoes are found in the Altiplano than in most other zones. The varieties of *Solanum ajanhuiri* have characteristics of tolerance to frost and drought similar to those of the *Solanum juzepczukii* but they do not have high glycoalkaloid contents and are therefore non bitter. At intermediate and lower altitudes the *Solanum tuberosum* ssp. *tuberosum* varieties are the most widespread whereas the other species predominate at altitudes higher than 3500 masl. Notwithstanding, recent findings showed that the actual upper limit for all potato varieties has increased to around 4300 masl [14] in response to increased temperatures and disease pressure at lower altitudes brought about by climate change.

The Andes represent the largest and highest mountain range in the tropics, and thus a suitable ecosystem to study changes in climate and how they affect the natural resources and the livelihood they sustain. Recent studies based on local meteorological networks [15], [16] have shown a significant warming trend after 1979 (0.32–0.34°C/decade). This warming trend shows some interesting features. Below 1,000 m there is a substantial difference between the eastern (Amazonian) and the western (Pacific watershed) slopes i.e. no warming trend on the eastern slope whereas on the western side there is a warming trend of 0.39°C/decade. On this flank, there is an almost linear decrease of the warming trend with altitude, reaching down to 0.16°C/decade above 4000 m. Thus there are differences between the Eastern and Western slopes and a definite vertical structure. This behavior differs from other mountain ranges in other latitudes e.g. European Alps and Tibet. A warming trend is also projected for the future [17], but differences as functions of position and altitudes are yet to be studied.

Summer frost events are caused by radiative cooling and are common throughout the Andean highlands and can occur at any time during the growing season [9], [18]. Frost can cause partial or complete loss of foliage, leading to a reduction in photosynthate production and hence yield. In turn, crop failure caused by frost damage may lead to a decrease in the total area planted to potato in the subsequent season due to seed shortage [18]. The temperature at which leafage frost damage occurs depends on the species and the cultivar. For *Solanum tuberosum* subsp. *andigenum*, frost damage is likely to occur when the temperature drops to 2°C or lower [19]. Higher frost resistance exists in other cultivars and in wild potato species. For example, cultivated potato species such as *S. ajanhuiri* and *S. curtilobum* are damaged at −3 to −5°C, whereas *S. juzepzuckii* generally resists temperatures down to −5°C and perhaps even lower [20].

Crop growth simulation models can be used to analyze constraints and opportunities for crop yields in complex production systems (e.g. 4,5). One of the limitations for using simulation models in this context is that most available models are calibrated only for varieties of *S*. *tuberosum* subspecies. Almost all modeling work has been done for so-called ‘modern’ varieties from formal breeding programs; with a few exceptions [21]. This can be particularly problematic when working in areas of high genetic diversity, where landraces with rather distinct growth characteristics may coexist with modern cultivars. A more in-depth understanding of the most important crop growth limiting factors as affected by genetic diversity is needed to arrive at more robust recommendations to reduce the vulnerability and improve the productivity of potato in environmentally challenged complex cropping systems. Comparative growth analysis and its translation into potato growth models can constitute a useful tool for scientists, particularly under climate variability and global climate change conditions in areas of rich genetic diversity.

The objective of the present study was to conduct a comparative growth analysis of four contrasting genotypes from the species *S. juzepczukii*, *S. ajanhuiri* and *S. tuberosum* subspecies *andigenum* and *tuberosum* and translate it into a modeling of their agronomic response. It included assessing their performance under contrasting agroecological conditions in the high Andes as well as the impact of frost events.

## Materials and Methods

This study did not involve neither human nor animal subjects. Field experiments were conducted in research stations and farmer fields and no law regulated permits were required because all materials used are commonly used by farmers with no environmental negative impacts. No protected species were sampled.

Permission to work on PROINPA’s field research sites (Patacamaya 2, Patacamaya 3, Puchuni and Laurani) was granted by Mr. Enrique Carrasco, then Leader of PROINPA.

Permission to work on farmers’ fields was granted by Mr. Vitaliano Mamani, Mrs. Maria Laura and Mr. Juan Carlos Huanca, under a collaboration agreement with PROINPA.

All authorizations were granted as verbal agreements on a *bona fide* basis.

### Germplasm

Four contrasting germplasm (species, cultivars, varieties, and landraces) - three native landraces and one introduced cultivar - representing the genetic diversity cultivated in the high Andes were studied. The four genotypes were Bola luki, referred to as Luki in this paper, a bitter potato variety of *Solanum juzepczukii* (3× or triploid); Chiar ajanhuiri, referred to as Ajanhuiri, a diploid (2×) variety of *Solanum ajanhuiri*; Gendarme, a tetraploid (4×) variety of *Solanum tuberosum* ssp. *andigenum*; and Alpha, a European tetraploid variety of *Solanum tuberosum* ssp. *tuberosum* [7], [22].

### Experiments and Data Collection

Four field trials, comparing four potato genotypes, were carried out in farmer's fields or on experimental stations in different locations (Patacamaya 2, Patacamaya 3, Puchuni and Laurani) in high and semi-arid zones in Bolivia (Table 1), where potato is the dominant crop, grown as the first crop after a fallow period. Within each site, complete randomized block designs with 3 or 4 replications were implemented in plots of 25.2 m^2^. The planting density was 4.76 plants/m^2^, a density suitable for the potato plow used in the zone. The tuber seeds used were from certified quality and were stored homogeneously during five months before planting. In addition, another experiment was conducted at Wichukollu locality, where the effect of frost (−2.51°C befall at 94 days after planting) on the four studied varieties was assessed.

**Table 1:** General description of the locations where the experiments were conducted.

	Latitude	Longitude	Altitude	Year	Rainfall	Minimum	Maximum	PAR	Soil	
Location	S	W	(masl)		(mm)	Temp (°C)	Temp (°C)	(MJ m^−2^d)	texture	
Patacamaya 2	17°14′	67°55′	3789	1998	437	4.6	18.7	10.4	SaCL	
Patacamaya 3	17°16′	67°55′	3789	1998	350	4.6	18.7	10.4	SaCL	
Puchuni	17°16′	68°13′	3950	1998	513	2.4	17.4	10.3	SiL	
Laurani	17°14′	68°11′	3850	1999	430	3.3	16.8	10.3	CL	
Wichukollu	17°01′	68°05′	3911	1999	445	3.0	16.5	10.1	CL	

Where: C is clay, L is loam, Sa is sand and Si is silt. Temperatures and PAR are monthly values and rainfall is cumulated value during crop cycle.

Plots were homogeneously managed to assure non-limiting factors for achieving potential production. Based on previous fertilization trials in the region [23], N was applied at 60–80 kg/ha and P_2_O_5_ at 100–120 kg/ha, in addition to 5 t/ha of bovine manure. Potassium was not applied due to the high levels of this element in these soils. Crops were rain-fed, with supplemental irrigation to bring the soil to field capacity when soil moisture dropped below 75% of field capacity, as determined by weekly soil samplings. Ridomil MZ (metalaxyl-M and mancozeb) and Bravo 500 (chlorothalonil) were used against potato late blight (caused by *Phytophthora infestans* Mont. de Bary). Synthetic pyrethroids, Karate (lambda-cyhalothrine) and Lorsban (chlorpyrifos) were used to control Andean potato weevils (*Premnotrypes latithorax* Piercei, *P. solaniperda* Kuschel, and *Rigopsidius tucumanus* Heller) and potato tuber moths (*Phthorimaea operculella* Zeller, *Symmetrischema tangolias* Geyen and *Paraschema detectendum* Povolny).

Precipitation, minimum and maximum temperatures were measured in the field using Hobo data loggers (ONSET Pro Series H08-032-08, USA). Global solar radiation was measured at meteorological stations representative of the experimental sites. Photosynthetically active radiation (PAR) was calculated as half of the global solar radiation [24].

During the crop growing period, plants were sampled three to five times from the plots. Intermediate harvests consisted of 4 plants per plot, while the final harvest consisted of 20 plants. Biomass was divided into leaves, stems, and tubers. Roots were not measured since they are difficult to collect. For each biomass group, total fresh weight was determined and their dry matter fraction estimated using samples of 150 g fresh weight subsequently dried at 75°C until constant weight which occurred after 48 to 72 hours.

The fraction of green canopy cover was estimated in triplicate in each experimental unit, every first or second week, from emergence to the final harvest. A wooden frame of 70 cm×90 cm divided into 100 cells of 7 cm×9 cm was used. The frame covered the area of three plants and cells were marked as covered if leaf surfaces occupied more than 50% of the cell area.

### Data Processing

The analysis of variance for the comparative growth analysis of all four genotypes was conducted by pooling the results from the four locations, once the homogeneity of the variance across experiments was demonstrated with the Bartlett test. The crop growth responses tested included: Maximum Canopy Cover (MCC), Light Use Efficiency (LUE), Dry Tuber Yield (DTY) and Harvest Index (HI). A summary of the variables and parameters of this study is described in Table 2.

**Table 2:** Abbreviations, list of variables and parameters used in the article.

Abbreviation	Description	Units
MCC	Maximum canopy cover	Fraction
LUE	Light use efficiency	g MJ^−1^
DTY	Dry tuber yield	t ha^−1^
HI	Harvest index	%
PAR	Photosynthetically active radiation	MJ m^−2^
Fo	Initial fraction of light interception	Fraction
Ro	Relative rate of light interception increase	°C^−1^d^−1^
D	Twice the duration from t_0.5_ to the end of the light interception	°Cd
t_0.5_	Time when the fraction of light intercepted is reduced to 50% of its Maximum value attained at MCC	°Cd
TIO	Tuber initiation onset	°Cd
TM	Tuber growth cessation or maturation	°Cd
X_0_	Point in thermal time when the maximum speed of translocation of assimilates is given	°Cd
TG_max_	Maximum tuber growth or bulking rate	g m^−2^ °Cd^−1^

The linear additive model used was:
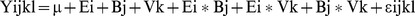
Where



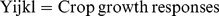


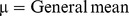


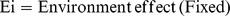


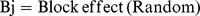


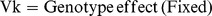


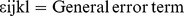



#### Growth analysis

Field measurements of plant growth components such as leaf area and the weight of plant parts provided for the parameters required for growth analysis. Maximum canopy cover MCC was calculated from the observed data through regression analysis. Growth parameters such as the rate of relative increase of light interception (Ro), the initial fraction of light interception at plant emergence (Fo), and the time when the fraction of light intercepted was reduced to 50% (t**_0.5_**) were estimated using curve fitting. The tuber initiation onset (TIO), the maximum tuber growth or bulking rate (TG**_max_**), and cessation of tuberization (TM) were calculated by fitting a logistic function (Sigma Plot V.9, USA) to the measured tuber weight as a function of thermal time (°Cd). The average LUE was determined as the slope of the curve between cumulative total dry matter and intercepted PAR [24].

#### Model description and calibration

Results from growth analysis can be integrated into mathematical models of the growth process, which constitute a robust tool for yield forecasting. This basic approach was followed in the present study to assess the response of the four potato genotypes and the results were used to calibrate the mathematical equations included in a slightly modified version of the LINTUL-potato model [24], [25], [4]. The model’s routines were originally developed in Visual Basic of Microsoft Excel and, once validated, programmed in C++. The model operates on a daily time step and its equations and parameters are described in [21]. The results of two experiments (Patacamaya 2 and Puchuni) were selected to calibrate the model and the results of the remaining two experiments were used for validation.

For modeling purposes, single plot variety mixtures were constructed, based on common practice by farmers. Our simulated mixtures included 65% Gendarme, 25% Luki, 7% Ajanhuiri and 3% Alpha [11], [13]. The yields from the mixtures in single-plots were compared against monoculture yield for each variety.

#### Model validation

Data sets from trials conducted in the other two independent sites (Patacamaya 3 and Laurani) were used to validate the calibrated model. Simulated data for MCC and DTY were compared with field results. Mean Bias Error (MBE), the Root Mean Square Error (RMSE), the Nash coefficient (Nash), and R^2^ [26], [27] were used to test the suitability of the calibrated model to simulate the growth of different genotypes under the experimental conditions.

#### Regression diagnostics of modeling

Regression diagnostics is the general class of techniques for detecting problems - with either the model or the data set - in regression analysis [28]. We adapted the residual analysis to evaluate simulated canopy cover data in time. This analysis was portrayed by plotting the phenological time series and residual values for detecting problem areas with outliers. The Durbin-Watson test was used to detect the presence of serial correlation in the residuals and influence statistics of points was measured through Cook’s D, DFFITS, DFBETAS and COVRATIO statistics [28]. The time series plotting was made in Sigma Plot V.9 software and the statistical analysis in SAS V.8.

#### Scenario analysis

*What if* type scenarios were constructed based on the validated model into which a frost routine, based on the data coming from the Wichukollu experiment, was incorporated to estimate potato yield losses for every genotype [29], [4]. Two issues were addressed with the scenario analysis: 1) How would different genotypes behave when exposed to progressive frosts (increasing by −1°C) during any of the four critical (30, 60, 90 and 120 DAE) phenological phases? 2) How would mixtures of varieties behave under extreme frosts, of frequent occurrence in the high Andes (−3°C at 60 and 90 DAE), compared to the monocultures? The model was set to simulate expected impact of progressive and extreme frosts on yield and it was compared to the production without frost stress.

## Results and Discussion

### Growth Analysis

Harvest index was the only response variable affected (P<0.05) by location (Table 3). Average LUE, MCC and DTY did not differ (P>0.05) among locations. The highest HI was recorded in Puchuni whereas the lowest HI occurred in Patacamaya 2. The average yield across all locations was six to sevenfold higher than the national average.

**Table 3:** Mean and standard error of the mean of potato growth responses to changes in environment and genotypes.

Environment	LUE^ns^	SE	HI*	SE	MCC ^ns^	SE	DTY ^ns^	SE
Patacamaya 2	2.658	0.082	79.19	0.97	72.69	5.60	10.98	0.84
Patacamaya 3	2.800	0.085	81.58	0.61	64.83	5.36	10.28	0.69
Puchuni	2.788	0.066	85.33	1.35	66.69	3.79	11.93	0.72
Laurani	2.529	0.147	84.12	1.62	69.58	4.87	9.29	0.58
Genotype	ns	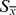	***	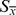	***	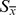	***	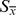
Alpha	2.687	0.091	86.55	1.55	40.05	1.18	7.21	0.38
Gendarme	2.661	0.104	83.13	1.17	84.21	2.76	13.07	0.78
Ajanhuiri	2.736	0.096	79.54	0.82	73.93	2.39	11.69	0.39
Luki	2.708	0.124	80.84	1.32	76.31	1.96	10.99	0.48

SE = Standard error of the mean; ns = non-significant P>0.05; ** = significant P<0.01; *** = significant P<0.001; Light Use Efficiency (LUE) in g DM MJ^−1^; Harvest Index (HI) in %; Maximum Canopy Cover (MCC) in %; and Dry Tuber Yield (DTY) in t ha^−1^.

MCC, DTY and HI differed (P<0.001) among genotypes (Table 3). Alpha showed the lowest MCC and the lowest DTY while Gendarme showed the highest MCC and the highest DTY. Ajanhuiri produced the lowest HI whereas the highest HI corresponded to Alpha. Luki gave intermediate values of MCC, DTY and HI, compared to the other genotypes evaluated. Changes in MCC explained 86% of the variation in DTY, whereas HI had no significant relationship with DTY (P>0.05). Average LUE did not differ (P>0.05) among genotypes.

The calculated values of the light use efficiency, (around 2.7 g DM MJ^−1^ as shown in Table 3) measured as the slope of the line describing the relationship between biomass production and absorbed PAR, were within the range of values reported in the literature for the *tuberosum* genotypes under temperate conditions [24], [30], [31].

Harvest index is generally regarded as the most important variable to estimate the productivity efficiency of the crops [32]. Generally the potato crop has higher HI values (about 0.80) than the other important crops (e.g. oil seeds 0.3 and cereals 0.5). In our case, HI was ≥0.8. However, in spite of the genetic diversity included in the study, the correlation with tuber yield was low, contrasting with literature findings [10]. This was probably due to MCC values that did not reach the level of a full coverage (MCC<1), which is likely due to the low F_0_ and slow R_0_ values in Andean genotypes, that might be explained by the low temperatures to which they are usually exposed during their growth in the Andean highlands. This poor value of the coverage level in the high Andes associated to its short duration contributes to a low biomass production [30], [33].

### Genotype by Environment Interaction

**Figure 1:**
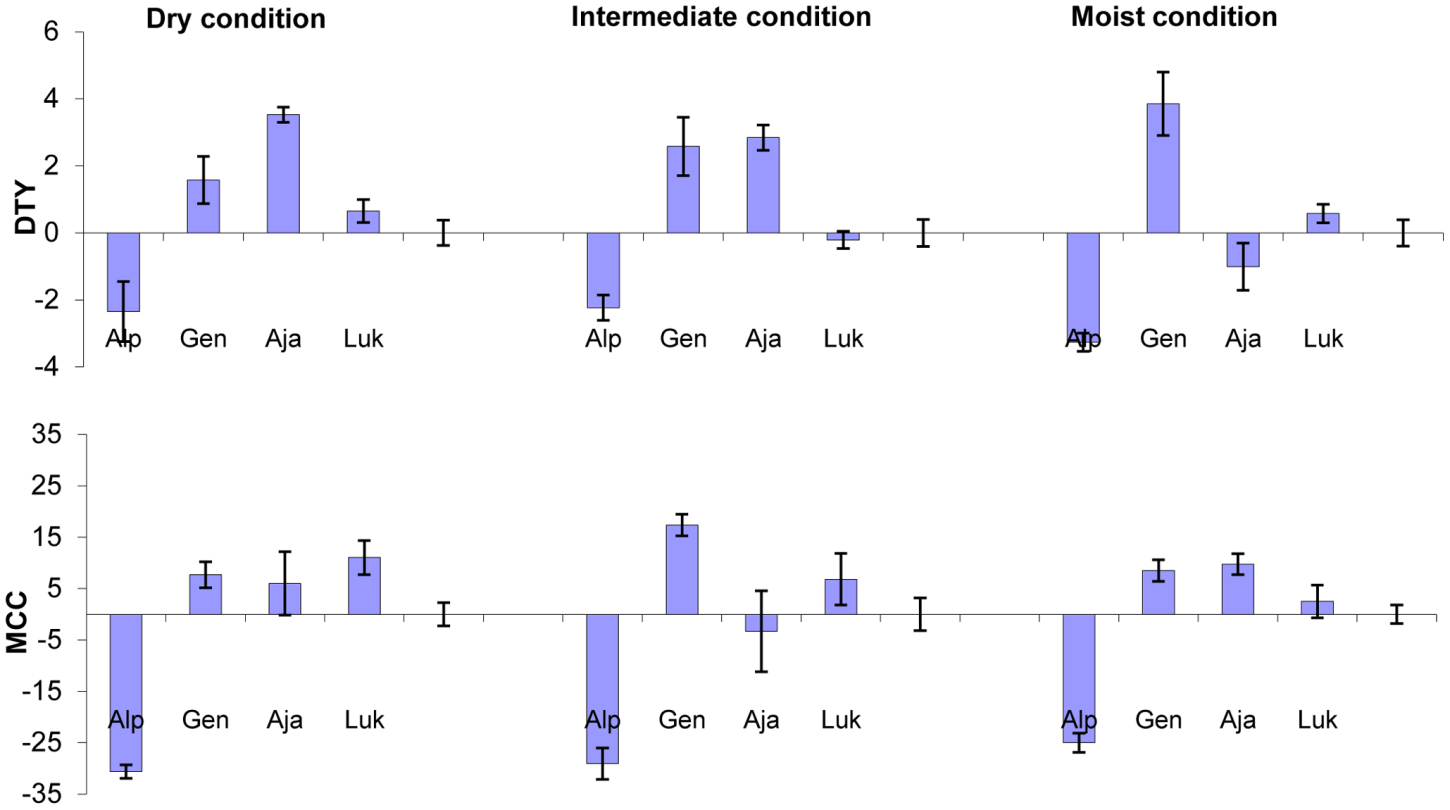
Genotype by Environment interaction for Dry Tuber Yield (DTY) and Maximum Canopy Cover (MCC) in three environment conditions in the high Andes. Residuals in Y axis, and standard error by variety.

DTY (P<0.01) and MCC (P<0.05) were significant for genotype x environment interactions. Figure 1, shows the performance of each genotype in the four experiments in which frost was not considered, against the average performance of all genotypes in each contrasting environment defined by cumulated rainfall as dry, intermediate and moist condition. Under all conditions, Gendarme outperformed the average yield whereas Ajanhuiri outperformed the average yield in dry and intermediate conditions only but was negatively affected by moist conditions. Alpha yielded much less than the average in all environments, a behavior explained by the overall low MCC. Luki was not influenced by environments and its yield was similar to the average.

### Modeling

The adapted LINTUL model adequately (R^2^>0.88) reproduced observed field responses in the calibration experiments (Table 4). Alpha showed the highest (5×10^−3^) light interception at emergence F_0_ value whereas the lowest value was shown by Luki (2×10^−3^). Rate of increase of light interception R_0_ was highest for Gendarme (7.3×10^−3^ °Cd^−1^), and lowest for Luki (5.5×10^−3^ °Cd^−1^). The duration of senescence (d) was longest for Alpha (approximately 38 days after the attainment of MCC) and shortest for Ajanhuiri (23 days following maximum canopy cover MCC). In the Bolivian Andes, Alpha is clearly an early maturing type [time of reduction to 50% of light interception t_0.5_ = 106 Days after Planting (DAE) and tuber initiation onset TIO = 24 DAE] while Luki is a very late maturing with a t_0.5_ of 120 DAE and TIO of 60 DAE (Table 4).

**Table 4:** Means of model parameters calibrated on four environments.

Genotype	F_0_***	SE	R_0_**	SE	d*	SE	t_0.5_***	SE	TIO***	SE	TM***	SE
Alpha	0.0050	0.0005	0.0068	0.0004	398	35	1163	27	259	17	748	48
Gendarme	0.0023	0.0003	0.0073	0.0006	268	25	1327	34	440	14	889	29
Ajanhuiri	0.0034	0.0004	0.0063	0.0003	253	43	1360	39	469	30	944	21
Luki	0.0020	0.0001	0.0055	0.0005	321	38	1329	50	650	29	984	56

About 11 degree days (°Cd) is equivalent to 1 day. SE = Standard error of the mean; * = significant P<0.05; ** = significant P<0.01; *** = significant P<0.001; F_0_: initial fraction of light interception; R_0_: rate of relative increase of light interception in °C^−1^d^−1^; d: twice the duration from t_0.5_ to the end of the light interception in °Cd; t_0.5_: time when the fraction of light intercepted is reduced to 50% of its maximum value attained at MCC (t_0.5_); TIO: tuber initiation onset in °Cd; and (TM): tuber growth cessation or maturation in °Cd.

The growth and developmental differences between Alpha and Luki genotypes is further evidenced by the time when maximum bulking rate TG**_max_** is attained; 60 DAE and 90 DAE for Alpha and Luki, respectively. The intercept and the slope were equal (P>0.05) to 0 and 1, respectively. The tuberization dynamics and parameter values for all genotypes are shown in Table 5.

**Table 5:** Coefficients of the logistic regression for dry matter translocation to tubers (HI, X_0_ and R^2^) and maximum tuber growth or bulking rate (TG_max_).

Genotype	HI	SE	X_0_	SE	R^2^	T_Gmax_	SE
Alpha	0.871	0.047	726.38	31.67	0.96**	1.50	1.18
Gendarme	0.814	0.029	874.92	20.50	0.99**	3.79	1.56
Ajanhuiri	0.808	0.043	959.44	25.60	0.99**	2.78	1.71
Luki	0.768	0.040	1030.25	25.27	0.97**	2.28	1.99

Where: HI is the maximum value that α reaches, X_0_ is the point in thermal time (°Cd), where the maximum speed of translocation of assimilates is given, TG_max_ is the Maximum tuber growth or bulking rate (g m^−2^ °Cd^−1^). SE is the Standard error; R^2^ is the determination coefficient at P<0.01 (**).

The R^2^ values (Table 6) show that the model explained more than 81% of the variations in DTY obtained experimentally for all varieties. For Luki and Ajanhuiri, the model explained more than 81 and 88% of the variation in the measured variable, respectively. For Alpha it explained more that 89% of the variation whereas for Gendarme, the model performed quite well, with a R^2^ value of 96% for DTY. All other statistical metrics used to test the validity of the model (MB, RMSE and Nash) to simulate potato yield and yield components under the conditions encountered in the high Andes also showed the model’s robustness (Table 6).

**Table 6:** Mean Bias Error (MBE), Root Mean Square Error (RMSE), Nash and R^2^ validation coefficients for Dry Tuber Yield.

DTY	Alpha	Gendarme	Ajanhuiri	Luki
MBE	0.0696	0.4678	0.8964	0.3564
RMSE	0.7386	0.8638	1.4764	0.9737
Nash	0.96	0.98	0.95	0.89
R^2^	0.89	0.96	0.88	0.81

**Figure 2:**
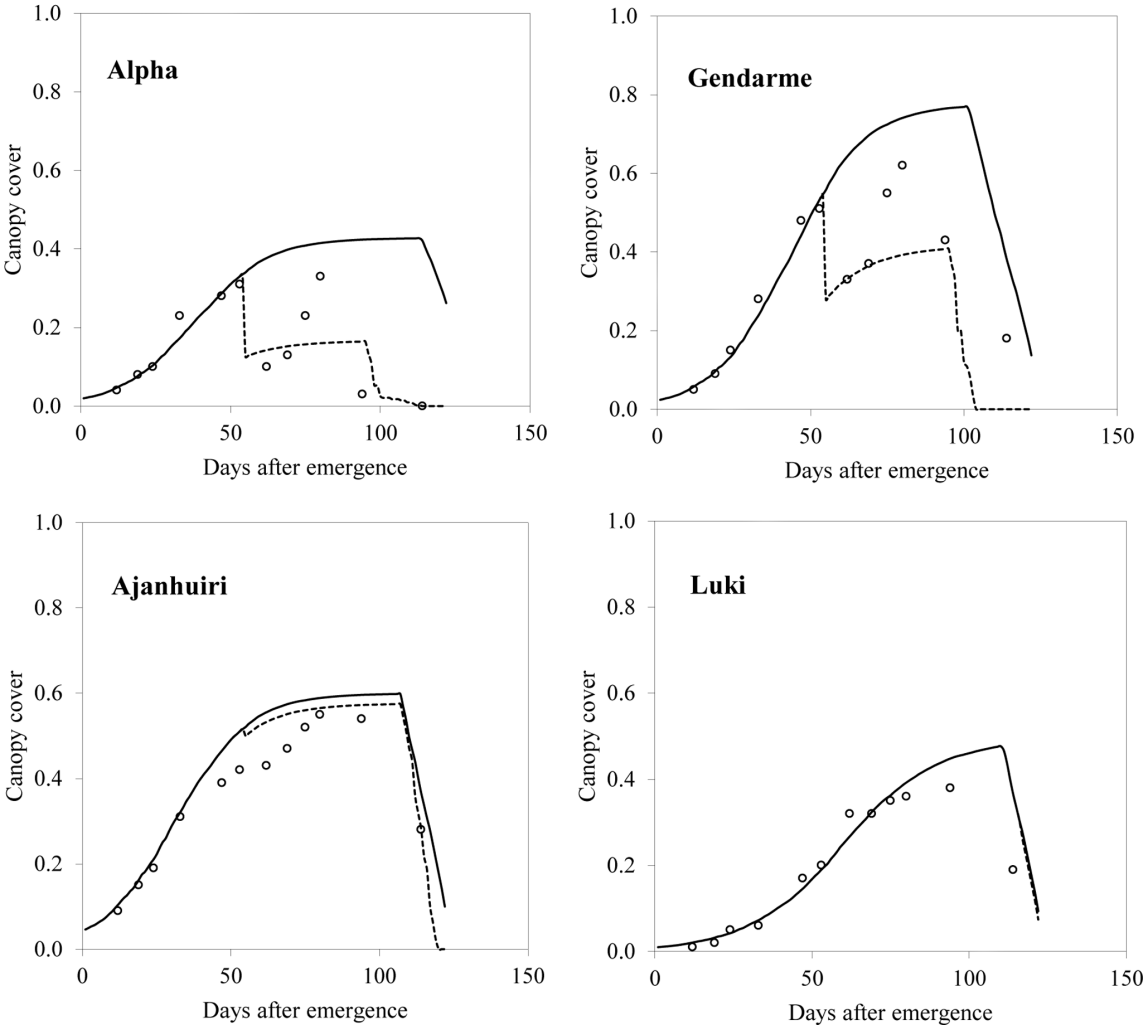
A frost (−2.51°C) impact on canopy cover at 94 days after planting in Wichukollu locality.

The *S. tuberosum* ssp. *andigenum, S. ajanhuiri* and *S. juzepczukii* seem to be well fitted for the harsh and variable environmental conditions prevalent in the high Andes, as suggested by all varieties. Concerning frost, differences in frost tolerance among the tested germplasm were evidenced by the differences in canopy cover retention following a frost episode. Figure 2 shows that Luki was not affected by frost; the effect on Ajanjuiri was minimal. On the other hand, Alpha was severely affected by the same frost episode. Nonetheless, genotypes from the subspecies *tuberosum* might play a role for specific objectives and climatic conditions, as suggested by Alpha performance under less stressful conditions. The genetic diversity of potatoes in the high Andes and their different responses to stresses suggests that potato production there can be highly increased with an adequate choice of germplasm, crop management and seed quality [34], [33].

Tuberization dynamics provides a clear differentiation among the genotypes studied [21]. Earliness defined as tuber initiation TIO is one of the growth parameters that express differences among genotypes. The earliness of Alpha was probably prompted by environmental conditions in the experimental sites where mean temperature was around 12°C with around 12 h of photoperiod as it has been reported that short photoperiods and low mean temperatures trigger an early onset of tuber growth and bulking in *S. tuberosum* ssp. *Tuberosum* [35].

It seems that the simultaneous occurrence of early tuber bulking and fast bulking rate traits is of high importance in the environmental conditions found in the high Andes. In spite of its earliness, the slow bulking rate of Alpha was associated with its comparative lower yields. On the contrary, Gendarme with its intermediate bulking initiation but fast-bulking rates produced the highest yield. Intermediate tuber initiation TIO combined with intermediate bulking rates produced intermediate yields.

Alpha accumulates assimilates earlier thus relying on a mechanism for escaping frost damage provided it happens late in the growing season. On the other hand, Luki presents a delayed TIO and a slow tuberization rate and thus extreme frost events (temp<−5°C) can disrupts the accumulation of assimilates and might cause losses.

The *juzepczukii* (var. Luki) and *ajanhuiri* genotypes outperformed the yield obtained with the mixtures under severe frosts. These results highlight the importance of the role played by local varieties tolerant to frost and drought stresses. Since the simulations were based on models parameterized with experimental results, yields obtained are feasible if good quality seeds and adequate management are implemented.

### Modeling Diagnostics

**Figure 3:**
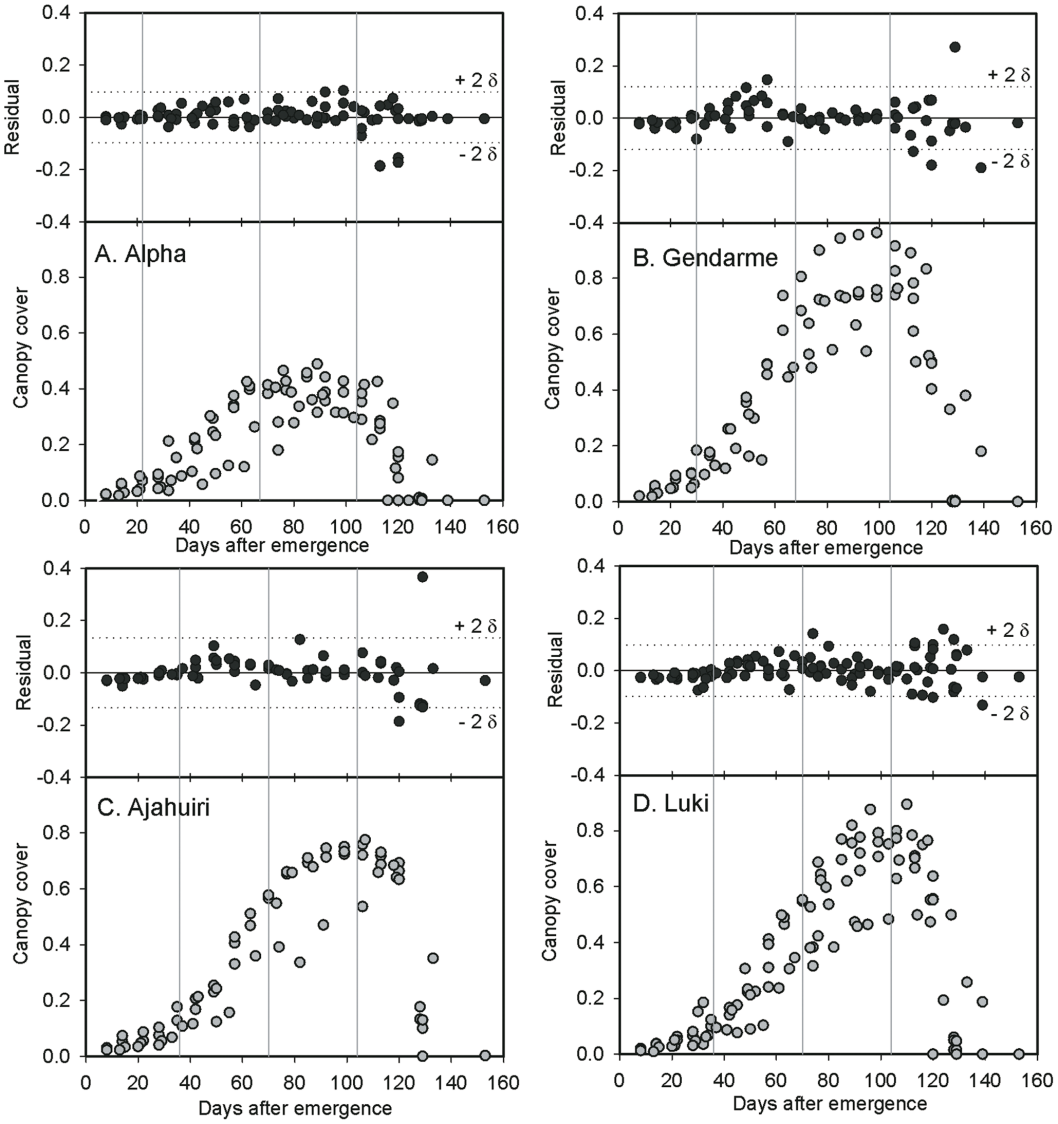
Residual dynamics analysis for Canopy Cover in Alpha, Gendarme, Luki and Ajanhuiri varieties.

The residual analysis showed a good spread of the values throughout the growth cycle. Luki presented a few points out of the accepted confidence envelop during the MCC and senescent phases. This was probably due to the rosette-type morphology which makes this variety susceptible to flattening (Figure 3).

For the emergence, maximum growth rate and MCC phases, the residual values were distributed within two standard deviations from the mean. Nonetheless, the regression diagnostics (Cook’s D, DFFITS, DFBETAS and COVRATIO), showed flagging points during senescence. This might be due to the lack of accuracy of the visual estimation of canopy cover, as the human eye cannot differentiate photosynthetically active leaves from senescent ones, particularly at the early stages of senescence. The use of digital cameras and appropriate segmentation techniques can reduce these problems (unpublished results from Production Systems and Environment subprogram at CIP).

### Scenario Analysis

**Figure 4:**
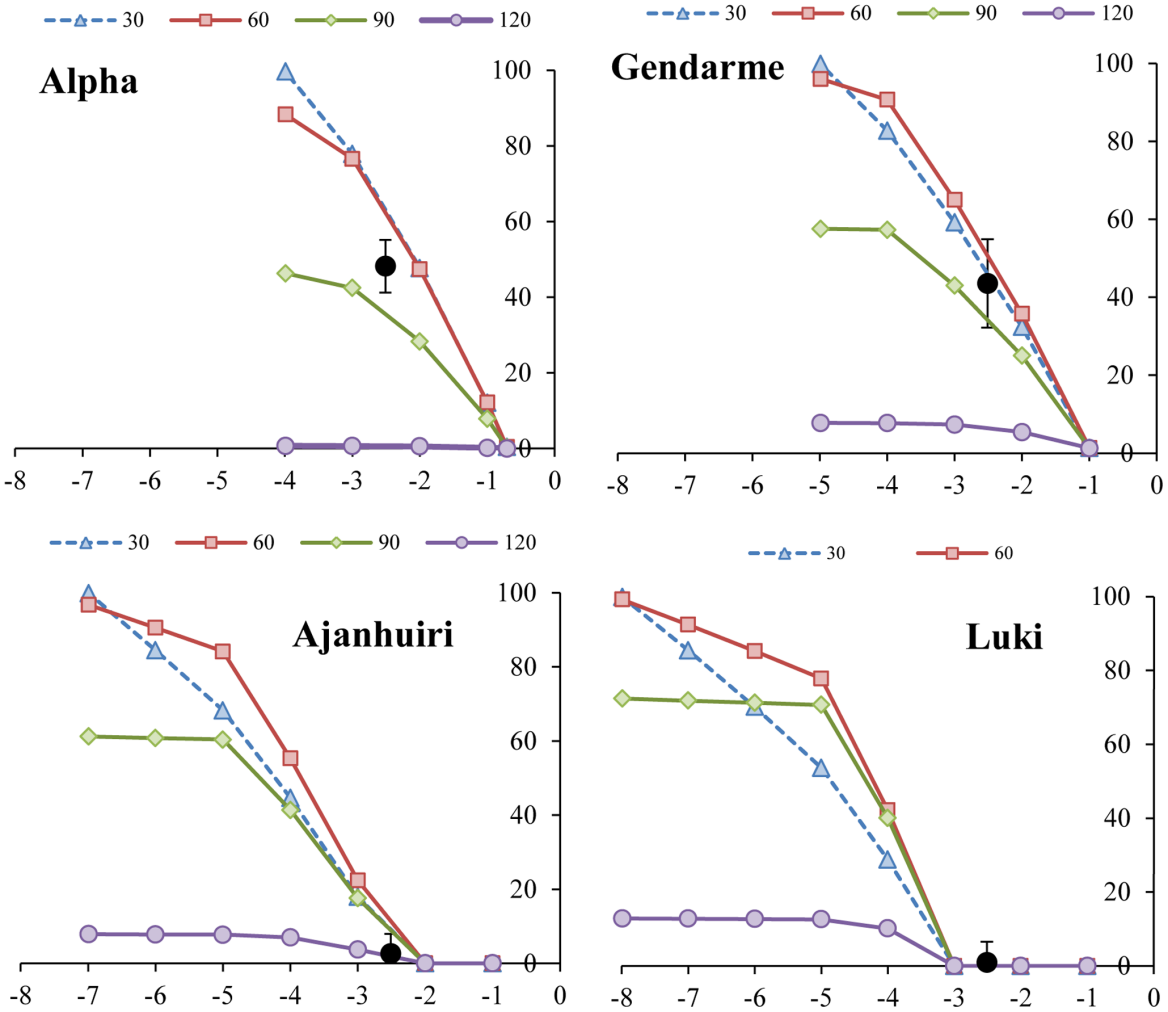
Percentage of tuber yield loss affected by frost (°C) at 30, 60, 90 and 120 days after planting on four potato varieties. In circles real losses on yield caused by frost (−2.51°C) occurred at 94 DAP.

The validated model was used to run *what if* type scenarios, to assess the likely impact of frost events - at specific phenologic stages – on yields. A threshold for absolute growth cessation with no regrowth was established in the model for each genotype (Alpha −4°C; Gendarme −5°C; Ajanhuiri −7°C; and Luki −8°C) occurring at different DAE. Overall, highest losses were evidenced for early frost events with average yield reductions ranging from 70 to 100% when occurring at 30 and 60 DAE. When the frosts were simulated at 90 DAE, the average reduction was around 50%. This late frost affected Luki the most with yield reductions of around 70%. In contrast, the early variety Alpha was the least affected by late frost with only about 40% tuber yield reduction. The latest simulated frost at 120 DAE caused minimal damages on tuber yields (<10%) in all genotypes (Figure 4).

**Figure 5:**
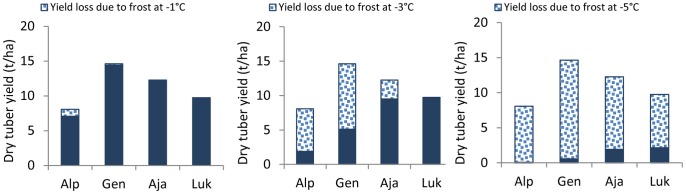
Impact on dry tuber yield loss due to frost at −1°C, −3°C and −5°C simulated at 60 days after emergence.

It is noteworthy that when the frost event occurs at 60 DAE, there is a drastic yield reduction in all genotypes. This seems to be associated to the fact that this is the time when canopy maximum growth rate and tuber initiation are also occurring. Alpha was the most affected with losses increasing exponentially as the severity of the frost augmented. Total loss in Alpha was caused by a −5°C frost. Gendarme presents a minimal damage at −1°C (0.2 t/ha), important losses at −3°C (9.5 t/ha) and a severe loss of tuber production at −5°C (14 t/ha). Ajanhuiri, in turn, was not affected at −1°C, presented a minor reduction at −3°C (2.7 t/ha) and an important reduction of tuber production at −5°C (10.3 t/ha). Luki was not affected at −1 and −3°C, but at −5°C was almost devastated (Figure 5).

We also simulated total yield comparing monoculture and single plot mixtures containing fractions of total acreage with four varieties distributed in the field (Alpha at 0.03, Gendarme at 0.65, Ajanhuiri at 0.07, and Luki at 0.25) the proportions mimicking the actual genotype distributions encountered in farmer fields. Scenarios without and with frost occurrence (−3°C at 60 and 90 DAE) were compared (Table 7). Frost free seasons resulted in average yields of 13 t/ha for the mixture. Gendarme in monoculture out yielded the simulated mixture. On the other hand, the mixture yielded 5.3 t/ha when affected by frost simulations. This yield was higher than the resulting from the commercial varieties Alpha and Gendarme but lower than those produced by Ajanhuiri and Luki as monocultures. If the tendency to a reduced planting of frost tolerant genotypes (Ajanhuiri and Luki) is confirmed, the production based on commercial potatoes can attain only 3.5 t/ha (Gendarme) when frosts occur (Table 7), thus local frost tolerant varieties provide an insurance under uncertain climate.

**Table 7:** Cumulative total dry yield (tons) under monoculture and mixture (Mix) conditions: without frost; with −3°C frost at 60 and 90 DAE.

Frost free						
Genotype	Monoculture					Mixture
Alpha	8.1	–		–	–	0.2
Gendarme	–	14.6		–	–	9.5
Ajanhuiri	–	–		12.3	–	0.9
Luki	–	–		–	9.8	2.4
Total	8.1	14.6		12.3	9.8	13.0
**Frost (−3°C at 60 and 90 DAE)**						
**Genotype**	**Monoculture**					**Mixture**
Alpha	1.5		–	–	–	0.0
Gendarme	–		3.5	–	–	2.3
Ajanhuiri	–		–	8.1	–	0.6
Luki	–		–	–	9.8	2.5
Total	1.5		3.5	8.1	9.8	5.3

Mixtures were composed of Alpha at 0.03, Gendarme at 0.65, Ajanhuiri at 0.07, and Luki at 0.25 of the land.

The varietal single plot mixtures used by the high Andes farmers seem to be a robust strategy to cope with climatic risks. This is particularly true under uncertain climate when a portfolio of options is warranted. The inclusion of native potatoes especially bitter varieties (*S*. *juzepczukii*) in the mix is a must under highly variable climatic conditions. The scenarios tested were purposely limited to fixed frosts at specific times during the growing period, just as examples of the modeling possibilities. The reality is that frosts are likely to occur randomly at different times thus highlighting the possible role of mixing varieties – as practiced for millennia by local farmers – to cope with those extreme events. Although the projection of future climate lean towards warmer nights, the increased variability of both night temperature and precipitation together with increased radiation cooling [17] warrant the inclusion of the drought and frost tolerance traits of *juzepczukii* and *ajanhuiri* genotypes. Therefore, the genetic diversity must be maintained since temperature and rainfall variability is expected to increase.

## Conclusions

The correspondence between the simulations and the results of the independent set of experiments conducted under various agroecological conditions demonstrated the adequacy of the model. This robustness applies not only to yield results under non-limiting factors but also to the forecast of the effects of frost events on canopy cover and correlated yield, which is a plus of the model. All statistical metrics used to test the validity of the model to simulate potato yield and yield components under the conditions encountered in the high Andes showed the model’s robustness.

The results indicate that Ajanhuiri and Gendarme varieties seem to be well suited for conditions with intermediate moisture levels, whereas, Ajanhuiri showed a better adaptation to drier areas. Ajanhuiri is a non-bitter variety tolerant to frost. Reintroducing good quality seeds into high Andean farming systems could enhance yields given its hardiness to cope with frequent frosts. For moister – rainfed or with strategic irrigation - areas the clear recommendation seems to be Gendarme and the like genotypes. Luki, on the contrary seems to be insensitive to environmental variations within the Altiplano.

Current conditions in the high Andes include a prolonged growing period of around 180 days. Small farmers diversify their risk through an assortment of genotypes in single plots. The observed diversity of tuberization dynamics of different genotypes supports this risk management strategy given the climatic and altitudinal variability and the low opportunity cost of the land. As the access to land decreases and direct as well as indirect effects of climate change impinge on the crop, new strategies must be sought. One such a strategy could be to develop genotypes with an early TIO and a very fast early bulking rate, able to escape from early biotic or abiotic shocks while sustaining a sizable yield.
